# Ability of the Sport Education Model to Promote Healthy Lifestyles in University Students: A Randomized Controlled Trial

**DOI:** 10.3390/ijerph20032174

**Published:** 2023-01-25

**Authors:** Chun-Chin Liao, Chien-Huei Hsu, Kuei-Pin Kuo, Yu-Jy Luo, Chun-Chieh Kao

**Affiliations:** 1 Office of Physical Education, Ming Chuan University, 5 De Ming Road, Gui Shan District, Taoyuan 333321, Taiwan; 2 Department of Child and Family Studies, Fu Jen Catholic University, 510, Zhongzheng Road, Xinzhuang Dist., New Taipei 242062, Taiwan; 3 Office of Physical Education, National Ping-Tung University of Science and Technology, 1 Shuefu Road, Neipu, Pingtung 91201, Taiwan

**Keywords:** physical education curriculum, health promotion, life satisfaction, interpersonal interaction

## Abstract

Although studies on sports performance, leadership abilities, group cohesion, and learning motivation have revealed that the sport education model contributes considerably to the development of healthy lifestyles, few studies have explored the development of healthy lifestyles from an educational intervention perspective. This study fills this gap in the literature. In addition, studies have mostly recruited elementary or middle school students; few have explored the effectiveness of sport education for college students. To fill this gap, this study conducted quasi-experimental research on university students by using different teaching strategies, with healthy lifestyles as the dependent variable. The research participants consisted of 95 students from Ming Chuang University distributed to an experimental group or control group. The experimental group was taught using the sport education model; the control group was taught using direct instruction. The results indicate that the sport education model has a stronger ability to promote healthy lifestyles than conventional teaching. Additionally, the results suggest that teachers should apply specific teaching strategies to cultivate and reinforce exercise habits and healthy behaviors among students. On the basis of the results, the researchers suggest that physical education teachers establish effective teaching strategies and promote healthy lifestyles to students.

## 1. Introduction

Studies have shown that physical education courses have a positive effect on youths’ health [1,2,3]. This has resulted in increased interest in the effect of physical activity on physical and mental health among educational personnel [4] and helped establish the key health benefits of physical education as the core objective of physical education courses worldwide [5]. Regular exercise habits developed through organized physical education courses have become a crucial part of the lifestyle of youths and have contributed greatly to the course learning outcomes [3]. Additionally, studies have indicated that physical education plays a vital role in value creation and improving mental health in students [6,7]. Studies have also revealed that regular physical activity can help individuals maintain a healthy lifestyle, help students develop skills, knowledge, and cognitive abilities [8,9], and improve mental health among youths [10,11,12]. The connection between health and physical activity has been widely acknowledged [13]. Accordingly, the literature on the evolution of educational frameworks for physical education has emphasized the necessity of a change in the priorities of physical education in school curricula [14,15].

Schools are widely considered the key institutions for promoting physical activity and exercise in youths [16]. Regular exercises can effectively reduce disease risks and mortality rates, thus benefiting physical health, mental health, and quality of life [17]. By conducting surveys and follow-ups on the exercise habits of individuals from adolescence to adulthood, one study discovered that lifestyle habits during university determine whether students commit to physical activity or sedentary behavior for the rest of their lives [9]. University physical education courses have a crucial influence on personal exercise habits and students’ physical education and exercise habits. Some studies have suggested introducing exercise interventions for young students to solve the public health problem of sedentary lifestyles and obesity [18]. The literature indicated that the prevalence of physical inactivity was 42.3% in Taiwanese adults [19]. A study conducted during the COVID-19 pandemic revealed that the overall prevalence of physical inactivity in China was 57.5%, and that in Wuhan was 63.5% [20]. This figure is four times higher than that prior to the pandemic and two times higher than the global average [21]; it is expected to increase if new waves of the pandemic occur. Therefore, the implementation of organized physical education courses during the pandemic is essential to ensuring students maintain regular exercise habits, develop healthy lifestyle habits, and receive proper health knowledge.

Medical, public hygiene, and education institutions have promoted physical training as a key strategy for reducing the high prevalence of physical inactivity [22]. Experts have suggested that students at least engage in the minimum level of physical education weekly to maintain their physical health [23]. Accordingly, physical education in schools plays a key role in promoting health. The games and competitive sports environment provided in physical education courses can increase the exercising capacity of students. Additionally, students are provided short, intermittent activities during these courses, which cause healthy physiological changes in students [24]. Miller [25] proposed that incorporating group activities into physical education courses and combining the activities with appropriate teaching strategies can improve the exercising capacity of students. This provides direct benefits to students’ health and also deepens their understanding of competitive sports [26]. Therefore, an increasing number of scholars have considered physical education crucial to promoting health and welfare in students [27,28,29].

Since the first promotion of the sport education model at an elementary school in the state of Ohio in the 1980s, the contemporary sport education model has gradually replaced conventional physical education courses, which involves students engaging in multiple activities [30]. The sport education model is a powerful teaching model [31] that has physical benefits if implemented correctly [32]. Students engaging in sport education courses can enjoy peer companionship and a sense of belonging because of high levels of tolerance from their peers [33]. In the sport education model, courses are held in the form of games to encourage exercise among students, provide students with comprehensive and positive exercise experiences, and enhance the course experience [34,35]. Fruitful sport education experiences during university positively affect the development of regular exercise habits in students after they enter the workforce. Most research on sport education models has centered on sports performance, leadership abilities, team cohesiveness, and learning motivation. Although studies on these topics have revealed that the sport education model contributes considerably to the development of healthy lifestyles, few studies have explored the development of healthy lifestyles from an educational intervention perspective. The first research motivation of this study is to fill this gap in the literature. In addition, studies have mostly recruited elementary or middle school students; few have explored the effectiveness of sport education for university students. Although the sport education model has garnered considerable attention, in the context of research continuity, room for further exploration remains. This is the second research motivation of this study. The main objective of this study is to fill the research gap regarding the sport education model. This study conducted quasi-experimental research on university students by using different teaching strategies, with healthy lifestyles as the dependent variable. The research findings are expected to fill the literature gaps and contribute to the theoretical and practical applications of the sport education model.

## 2. Methods

### 2.1. Research Participants

The research participants consisted of 95 students (47 men, 48 women) from Ming Chuang University. The participants were recruited from two classes and distributed to the experimental group (*n* = 49; age: 21.98 ± 0.80 years) and control group (*n* = 46; age 21.91 ± 0.96 years). The experimental group was taught using the sport education model; the control group was taught using direct instruction. Prior to data collection, the researchers obtained the consent form of the Institutional Review Board of National Taiwan University (serial number: 201812ES018).

### 2.2. Experiment Process

Limited by the teaching environment of the classroom system and a fixed class size, this study was unable to conduct randomized equal-group multifactor experiments. For this reason, an unequal-group pretest–posttest design was employed for the experiment. The experiment comprised pretest, intervention, and posttest stages. First, two classes taking a badminton course were recruited and screened using the inclusion criteria. Next, the classes were randomly assigned to the experimental or control groups and provided an explanation of the course framework. Finally, the participants’ consent was obtained.

In the intervention stage, participants were divided into the experimental or control groups. The participants underwent one session of a physical education class per week, with each session lasting 100 min, for 10 weeks. The intervention course was taught by a certified physical education instructor with more than 20 years of teaching experience. Both groups received the same physical education and sports technique guidance. However, the sport education model in the experimental group was incorporated as one of six course features. Participants were not permitted to change their exercise partners throughout the course.

### 2.3. Research Tool: Healthy Lifestyle Scale

Amended from the scale proposed in Chen et al. [36], the Healthy Lifestyle Scale was used to evaluate the students’ lifestyles through a questionnaire survey with 13 items. The items were divided into three dimensions, namely, health promotion, life satisfaction, and interpersonal interaction; the Cronbach’s α of the dimensions was 0.88, 0.86, and 0.69, respectively, indicating suitable construct reliability. The construct validity of the research tool was assessed. Factor analysis revealed that the Kaiser–Meyer–Olkin coefficients of the health promotion, life satisfaction, and interpersonal interaction dimensions were 0.87, 0.82, and 0.50, respectively, accounting for a cumulative explained variation of 60.6%. The scale was scored using a 5-point Likert scale (1 = strongly disagree; 5 = strongly agree). Examples of the items include “After the physical education course, I started participating in healthy outdoor activities,” “After the physical education course, I paid more attention to information and news related to health,” and “After the physical education course, I started to enjoy interacting with my classmates.” The external quality of the scale and the empirical data on the goodness-of-fit of the scale met the requirements for academic research.

### 2.4. Course Development and the Effectiveness of the Intervention Program

To ensure the effectiveness of the teaching intervention program, the principal investigator recruited one professional badminton instructor and two sport education scholars to convene a sport education model teaching design evaluation group. The group conducted two focus meetings on the intervention program, performed a concrete evaluation of the teaching design method and a substantive examination, and provided suggestions and feedback on the teaching process. The sport education model of the experimental group was incorporated into the six features proposed in Siedentop [37], namely, seasons, affiliation, formal competition, culminating events, record keeping, and festivity. The sport education model of the control group was designed by referencing the key points of the direct instruction approach highlighted in Pereira et al. [38], namely, (1) the teacher being the instructional leader of the unit and setting the learning goals and tasks; (2) the course being based on the repeated performance of specific sports techniques or skills; (3) students working cooperatively in groups of two or three to repeatedly practice the techniques specified for each learning task; student grouping throughout the class was variable across tasks; (4) the training content being indirectly applied to the context of the game; (5) the standard for determining student success being based on successful execution of the techniques; and (6) the teacher providing explanations to correct students’ mistakes. The educational objectives for the experimental group are listed in Table 1.

### 2.5. Statistical Analysis

Statistical analysis was conducted using the SPSS 19.0. The results were analyzed using five methods; (1) participants’ height, weight, and body mass index (BMI) were analyzed using descriptive statistics; (2) homogeneity in sex was tested using a chi-squared test; (3) homogeneity in the participants’ height, weight, and BMI was tested using independent sample *t*-tests; (4) after the effect of pretest scores was eliminated, the effect of the course on the healthy lifestyles of the experimental and control groups was analyzed using analysis of covariance; and (5) the effect size of the intervention was calculated.

## 3. Results

### 3.1. Homogeneity Testing

Analysis of the demographic statistics revealed homogeneity between the groups in terms of sex (χ^2^ = 0.097, *p* > 0.05), age (t = 0.365, *p* > 0.05), height (t = 0.905, *p* > 0.05), weight (t = 0.971, *p* > 0.05), and BMI (t = 2.828, *p* > 0.05). The participants’ demographic statistics are listed in Table 2.

The homogeneity of the within-class regression coefficients for the health promotion (F = 0.567; *p* = 0.454, η^2^ = 0.060), life satisfaction (F = 1.612; *p* = 0.207, η^2^ = 0.170), interpersonal interaction (F = 2.101; *p* = 0.151, η^2^ = 0.023), and overall healthy lifestyle (F = 1.188; *p* = 0.279, η^2^ = 0.013) variables were consistent with the hypothesis regarding the homogeneity of the within-class regression coefficient in the covariance analysis (Table 3). The data had high homogeneity and were ideal for experimental intervention. Given that the results supported the hypothesis, the data were suitable for covariance analysis.

### 3.2. Healthy Lifestyle Performance

The pretest and posttest scores of the two groups are listed in Table 4. For health promotion, the pretest and posttest scores of the experimental group were 3.09 (±0.56) and 3.45 (±0.44), respectively, and those of the control group were 3.06 (±0.54) and 3.25 (±0.61), respectively. For life satisfaction, the pretest and posttest scores of the experimental group were 3.39 (±0.63) and 3.69 (±0.49), respectively, and those of the control group were 3.41 (±0.68) and 3.46 (±0.59), respectively. For interpersonal interaction, the pretest and posttest scores of the experimental group were 3.72 (±0.76) and 3.95 (±0.72), respectively, and those of the control group were 3.79 (±0.61) and 3.57 (±0.73), respectively. For overall healthy lifestyle, the pretest and posttest scores of the experimental group were 3.28 (±0.50) and 3.60 (±0.38), respectively, and those of the control group were 3.28 (±0.50) and 3.36 (±0.52), respectively.

### 3.3. Covariance Analysis

The covariance analysis (Table 5) revealed that after the effect of the covariate (pretest scores) on the dependent variable (posttest scores) was eliminated, the adjusted statistics of overall healthy lifestyle (M = 3.60 > M = 3.36, F = 6.43, *p* < 0.05, η^2^ = 0.07), health promotion (M = 3.45 > M = 3.25, F = 3.26, *p* < 0.05, η^2^ = 0.03), life satisfaction (M = 3.69 > M = 3.46, F = 4.37, *p* < 0.05, η^2^ = 0.05), and interpersonal interaction (M = 3.95 > M = 3.57, F = 6.66, *p* < 0.05, η^2^ = 0.07) were statistically significant (*p* < 0.05). This indicates that the sport education model significantly affected the experimental group’s overall healthy lifestyle scores.

## 4. Discussion

### 4.1. Effect of the Sport Education Model on Healthy Lifestyle Performance

This study evaluated the effect of the sport education intervention on healthy lifestyles among students in terms of health promotion, life satisfaction, and interpersonal interaction. The preliminary results indicate that the sport education model has a stronger ability to promote healthy lifestyles than conventional teaching, which suggests the importance of further research on this topic. The sport education model is a crucial teaching strategy for promoting healthy lifestyles. The results corroborate those of studies specifying that the sport education model positively affects health promotion [42,43], life satisfaction [44], and interpersonal interaction [45,46,47]. Additionally, the results imply that teachers should apply specific teaching strategies to cultivate and reinforce exercise habits and healthy behavior among students [48,49,50]. In physical education, teachers encourage students to engage in physical activity, ensure students remain physically active and cultivate healthy habits, and emphasize key factors to developing active and healthy lifestyles [51]. The group exercise activities in the sport education model enable teachers to provide students with a realistic learning environment based on sport seasons in which students can play the roles of coaches or form competitive teams with shared goals. This enables students to control and lead the course, thereby creating satisfactory learning experiences [52,53]. On the basis of the results, the researchers suggest that physical education teachers establish effective teaching development strategies and promote healthy lifestyles to students on the courses.

Physical education should encourage physical activity in students, particularly for those who do not exercise regularly [54]. Schools have a crucial influence on physical health in youths [55]. As promoters of public health, physical education teachers play a crucial role in encouraging students to learn about healthy lifestyles [56]. In the sport education model, learning scenarios based on sports seasons provide students with various opportunities to engage in team-based exercises. Team affiliation prompts students to plan, practice, and compete together and provides them with opportunities to engage in community development activities as long-term members of a community. Thus, physical education courses based on the sport education model can help students contribute to achieving a goal. Scholars supporting the sport education model have proposed that sport education can be widely applied to exercise to promote health knowledge [57]. Exercise in the sport education model considerably improved most students’ (80%) health [43]. This may have also increased their willingness to continue playing sports into adulthood [7,58]. Sport education creates a win–win scenario for health promotion and physical education and can even encourage students to continue exercising after the course. Therefore, physical education must include health promotion. By facilitating the transfer of knowledge, encouraging team collaboration, and motivating students to learn about health, physical education courses can ensure that students develop lifelong exercise habits.

Individuals who are satisfied with life experience more positive emotions than negative [59]. The reinforcement of healthy habits is crucial to every stage of life; exercising both moderates and mediates life satisfaction [60]. Studies have reported that exercise capacity is closely related to life satisfaction [61]. In addition, exercise affects life satisfaction in youths. Participation in team sports is vital to life satisfaction because it helps them develop confidence [62,63,64,65,66]. Studies on sports have also demonstrated that participation in sports is positively related to the fulfilment of psychological needs, self-determination and motivation, positive emotions, and life satisfaction. Life satisfaction also has a positive relationship with exercise. Participation in sports has been demonstrated to decrease anxiety, depression, and negative emotions; increase self-esteem; improve cognitive function; and positively affect life satisfaction [67]. These results support those of Dhurup [68], who indicated that active, tolerant, and effective teaching methods in a high-quality physical education framework create a conducive environment for cultivating healthy and satisfying lifestyles. Students must be satisfied with their education to develop positive thinking abilities, achieve favorable learning outcomes, and cultivate healthy behavior. The sport education model provides teachers with an opportunity to design effective courses and enables students to roleplay and develop an interest in competitive sports. By emphasizing collaborative learning and teamwork, teachers can help students become leaders, form heterogenous groups for learning, and incorporate competition into group activities. Teachers can also hold celebratory events to create a positive atmosphere for the students. The sport education model can be implemented into physical education courses to ensure life satisfaction among youths, thereby improving their quality of life and health and encouraging them to maintain a healthy lifestyle.

Student interaction is a key feature of physical education courses [47]. The goal of youth development is individuality. The most crucial aspect of this process is the development of strong relationships with peers and personal and shared responsibilities, both of which are crucial to the development of social skills [40]. The results of this study are consistent with those of studies specifying that the sport education model encourages peer interaction among students and enables them to monitor their community interaction. The games in the sport education model encourage exercise and interpersonal interaction among students. In addition, the sports competitions help students devise strategies. During the games, students can act as referees, strengthening their decision-making skills. The interaction among students throughout the learning process is a key experience that contributes to creating a positive learning environment, which benefits students [35,40,69,70]. In the sport education model, students are given specific responsibilities and contribute to managing sporting events. The model encourages students to participate in sports and thereby creates opportunities for interaction and communication. In courses based on the sport education model, roles are distributed to team members to push students to participate in physical activity, thereby encouraging interaction and communication. By playing various roles, students can develop a positive relationship with exercise, learn new skills, strengthen their decision-making abilities, become more adaptive, and learn to take responsibility, thereby engaging in a fulfilling learning experience. Interpersonal interaction is a key benefit of the sport education model. In addition to improving the health and physical fitness of students, physical education courses should help students develop interpersonal relationships.

Given that the ultimate goal of physical education is to improve health and welfare, physical education must improve the social and emotional skills of students, urge students to lead healthy lifestyles, and emphasize the importance of public hygiene. The sport education model is based on games, which play a crucial role in physical education. Games can ensure that students are satisfied with physical education, help students develop skills, allow students to enjoy relaxed gaming environments, and instill in students the value of interpersonal interaction. This quasi-experimental study revealed the ability of the sport education model to cultivate healthy lifestyles in students. The results indicated that the sport education model is effective. The students who participated in the physical education course based on the sport education model exhibited healthy lifestyles. These results provide concrete evidence for teachers of physical education interested in using teaching methods based on the sport education model to promote healthy lifestyles in students.

### 4.2. Research Limitations

This study had several limitations. First, this study did not use a randomized equal-group multifactor experimental design. As a result, the results may have been affected by sampling bias (e.g., the experience and team participation) and thus cannot be completely generalized to students of other countries, regions, or educational backgrounds. Second, the teaching intervention was highly structured and supportive because of the students’ self-efficacy. Finally, the research participants consisted only of students from one university, and the course framework was solely based on badminton. Researchers should exercise caution when generalizing the results to courses based on other sports.

### 4.3. Research Suggestions

The results provide a solid foundation for research on exercise and physical education courses. Researchers should follow-up their participants for 1 or 2 months to determine whether the sport education model has a lasting effect in terms of encouraging healthy lifestyles. In addition, studies can use other indicators of the effectiveness of the sport education model. To explore the model’s ability to promote healthy lifestyles in university students, this study used indicators of healthy lifestyles. Studies can use students’ ability to maintain regular exercise habits and the effect of teams on individuals as indicators.

## 5. Conclusions

This study provided preliminary evidence indicating that incorporating the sport education model into physical education courses promotes healthy lifestyles in students and fills the gap in the literature regarding the sport education model. Organized teaching strategies for physical education courses are crucial to helping youths learn about health. The results can be of use to medical and healthcare providers. Organized physical education scenarios are crucial to preventing sedentary lifestyles. This study provides strong evidence of the ability of the sport education model to promote healthy lifestyles in university students. The results can be applied to other physical education activities to further promote healthy lifestyles in students. Teachers of physical education courses should integrate the sport education model into their courses. Considering the close relationship between youth and adult health, improving youth health through organized physical education strategies is essential.

## Figures and Tables

**Table 1:** Designing learning materials for educational objectives.

Feature	Teachers	Students	Educational Objectives
Seasons	(1) Arranging sports seasons and training (2) Adjusting time based on teaching items and content	(1) Learning about sports experiences (2) Gaining sports knowledge (3) Adjusting strategy and application	Cognitive objectives: (1) Game planning and management (2) Sports appreciation (3) Record keeping (4) Strategy implementation 2. Affective objectives: (1) Rational decision making (2) Etiquette, history, and rules of games (3) Team collaboration (4) Interpersonal interaction and team adaptation 3. Psychomotor domain: (1) Sport techniques (2) Physical fitness (3) Flexible rules that adapt to the ability levels of students
Affiliation	(1) Heterogeneous grouping (2) Conducting all practices and games in teams	(1) Assigning roles: team captain, referees, and record keepers (2) Planning jobs and participating in games	
Formal Competition	(1) Disclosing the event schedule (2) Disclosing rewards (3) Arranging warm-up matches	(1) Learning about teamwork (2) Conducting strategy drills and developing team chemistry (3) Appreciating the game	
Culminating events	(1) Disclosing event schedules for the finals (2) Analyzing the competition in the finals	(1) Conducting strategy drills and developing team chemistry (2) Appreciating the game	
Record Keeping	(1) Instruction on the record-keeping method (2) Teaching the basis for final evaluation	(1) Learning the record-keeping method (2) Learning the evaluation method	
Festivity	(1) Maintaining a joyful atmosphere in classes (2) Hosting the awards ceremony in class	(1) Experiencing the meaning of sports (2) Appreciating the ceremony	

Organized by the author according to Jewett et al. [39], Kao [40], Siedentop [41].

**Table 2:** Demographic statistics of participants.

Variable	Experimental Group (*n* = 49)	Control Group (*n* = 46)	Significance
Gender (male; Female)	25:24	22:24	x^2^ = 0.097, *p* > 0.05
Age (years)	21.98 (0.80)	21.91 (0.96)	t = 0.365, *p* > 0.05
Height (M)	1.67 (0.09)	1.65 (0.09)	t = 0.905, *p* > 0.05
Weight (Kg)	62.41 (10.17)	64.37 (9.52)	t = 0.971, *p* > 0.05
BMI (kg/m^2^)	22.18 (2.09)	23.42 (2.19)	t = 2.828, *p* > 0.05

**Table 3:** Homogeneity of within-class regression coefficients.

Variables	TypeIIISS	df	MS	F	*p*	E.S.
Health promotion Error	0.158	1	0.158	0.567	0.454	0.060
	25.431	91	0.279			
Life satisfaction Error	0.470	1	0.470	1.612	0.207	0.170
	26.518	91	0.291			
Interpersonal interaction Error	1.044	1	1.044	2.101	0.151	0.023
	45.230	91	0.497			
Health lifestyle Error	0.248	1	0.248	1.188	0.279	0.013
	19.026	91	0.209			

Adjusted R^2^ (0.014, 0.032, 0.117, 0.048).

**Table 4:** Pretest and posttest performance for each variable.

Variables	Control Group		Experimental Group	
	Pretest	Posttest	Pretest	Posttest
Health promotion	3.06(0.54)	3.25(0.61)	3.09(0.56)	3.45(0.44)
Life satisfaction	3.41(0.68)	3.46(0.59)	3.39(0.63)	3.69(0.49)
Interpersonal interaction	3.79(0.61)	3.57(0.73)	3.72(0.76)	3.95(0.72)
Health Lifestyle	3.28(0.50)	3.36(0.52)	3.28(0.50)	3.60(0.38)

**Table 5:** Covariance analysis results.

Variables	TypeIIISS	df	MS	F	*p*	E.S.
Health promotion Error	0.91	1	0.91	3.26 *	0.04	0.03
	25.59	92	0.28			
Life satisfaction Error	1.28	1	1.28	4.37 *	0.04	0.05
	26.99	92	0.29			
Interpersonal interaction Error	3.35	1	3.35	6.66 *	0.01	0.07
	46.27	92	0.50			
Health lifestyle Error	1.35	1	1.35	6.43 *	0.01	0.07
	19.27	92	0.21			

* *p* < 0.05, adjusted R^2^ (0.019, 0.026, 0.106, 0.046).

## Data Availability

Data are available from the corresponding author upon reasonable request.
